# Ralaniten Sensitizes Enzalutamide-Resistant Prostate Cancer to Ionizing Radiation in Prostate Cancer Cells that Express Androgen Receptor Splice Variants

**DOI:** 10.3390/cancers12071991

**Published:** 2020-07-21

**Authors:** Carmen A. Banuelos, Yusuke Ito, Jon K. Obst, Nasrin R. Mawji, Jun Wang, Yukiyoshi Hirayama, Jacky K. Leung, Teresa Tam, Amy H. Tien, Raymond J. Andersen, Marianne D. Sadar

**Affiliations:** 1 Department of Genome Sciences, British Columbia Cancer, 675 West 10th Avenue, Vancouver, BC V5Z 1L3, Canada; abanuelos@bcgsc.ca (C.A.B.); yitou1@yokohama-cu.ac.jp (Y.I.); jobst@bcgsc.ca (J.K.O.); nmawji@bcgsc.ca (N.R.M.); jeanwang@bcgsc.ca (J.W.); ukiyoc@gmail.com (Y.H.); jleung@bcgsc.ca (J.K.L.); ttam@bcgsc.ca (T.T.); atien@bcgsc.ca (A.H.T.); 2 Department of Chemistry, University of British Columbia, Vancouver, BC V6T 1Z1, Canada; raymond.andersen@ubc.ca; 3 Department of Pathology and Laboratory Medicine, University of British Columbia, Vancouver, BC V6T 1Z7, Canada

**Keywords:** prostate cancer, DNA repair, AR-V7, ralaniten, EPI-002, enzalutamide, radiotherapy

## Abstract

Blocking androgen receptor (AR) transcriptional activity by androgen deprivation therapy (ADT) improves the response to radiotherapy for intermediate and high risk prostate cancer. Unfortunately, ADT, antiandrogens, and abiraterone increase expression of constitutively active splice variants of AR (AR-Vs) which regulate DNA damage repair leading to resistance to radiotherapy. Here we investigate whether blocking the transcriptional activities of full-length AR and AR-Vs with ralaniten leads to enhanced sensitivity to radiotherapy. Combination therapies using ralaniten with ionizing radiation were evaluated for effects on proliferation, colony formation, cell cycle, DNA damage, and Western blot analyses in human prostate cancer cells that express both full-length AR and AR-Vs. Ralaniten and a potent next-generation analog (EPI-7170) decreased expression of DNA repair genes whereas enzalutamide had no effect. FACS analysis revealed a dose-dependent decrease of BrdU incorporation with increased accumulation of γH2AX with a combination of ionizing radiation with ralaniten. An additive inhibitory effect on proliferation of enzalutamide-resistant cells was achieved with a combination of ralaniten compounds with ionizing radiation. Ralaniten and EPI-7170 sensitized prostate cancer cells that express full-length AR and AR-Vs to radiotherapy whereas enzalutamide had no added benefit.

## 1. Introduction

Radiotherapy is one of the primary treatment options for localized prostate cancer and when combined with androgen deprivation therapy (ADT) there is significant improvement in disease-free and overall survival for intermediate and high-risk disease [1,2,3,4]. Unfortunately, in spite of this improvement, up to 50% of high-risk patients will have biochemical recurrence [5] which is associated with poor prognosis [6]. Efforts to improve outcomes for these patients have revealed that the androgen receptor (AR) regulates DNA damage repair (DDR) in prostate cancer [7,8,9,10].

AR is a ligand-activated transcription factor comprised of a C-terminal ligand-binding domain (LBD) linked to the DNA-binding domain by the hinge region. The N-terminal domain (NTD) contains all of the AR’s transcriptional activity within its activation function-1 (AF-1) region. AR regulates the expression of many genes in prostate cells including those controlling cellular proliferation and survival thereby providing the rationale for ADT to block AR signaling as a systemic therapy for prostate cancer. ADT reduces the levels of androgens and includes orchiectomy, LH-RH agonist/antagonists and also the CYP17 inhibitor abiraterone acetate. Nonsteroidal antiandrogens such as enzalutamide and the other “-lutamides” compete with androgen for binding in the ligand-binding pocket in AR-LBD to reduce its transcriptional activity. Both ADT and antiandrogens are AR-LBD inhibitors and provide their therapeutic response by blocking AR signaling, but these therapies are not curative. Resistance to ADT and antiandrogens results in lethal castration-resistant prostate cancer (CRPC) by mechanisms that include: Expression of constitutively active truncated AR splice variants (AR-Vs) that lack an LBD; de novo biosynthesis of androgens; and gain-of-function point mutations in the AR-LBD. Targeting the Achilles heel of the AR, the NTD, with the ralaniten (EPI-002) compounds blocks all of these mechanisms of resistance to inhibitors of AR-LBD (ADT, abiraterone, antiandrogens) [11,12,13,14]. A Phase 1 clinical trial with the first generation antagonist to AR-NTD (ralaniten–acetate, previously known as EPI-506), showed some efficacy thereby providing proof-of-concept for targeting the AR-NTD in heavily pretreated CRPC patients that had failed enzalutamide and/or abiraterone and provided support for the ralaniten chemical scaffold [14,15]. A more potent and metabolically stable next-generation analog of ralaniten is starting clinical trials June 2020 for patients with CRPC (NCT04421222). 

Radiotherapy mediates a therapeutic response by inducing DNA double-strand breaks (DSB) that results in cell death when DSBs are not repaired and the cell continues to progress through the cell cycle. The most prominent arrest of most cancer cells in response to IR is in the G2 phase [16]. Most DSBs are repaired mainly by non-homologous end-joining (NHEJ) and homologous recombination (HR). NHEJ is dominant in G0/G1 and G2 of the cell cycle but can occur throughout the cell cycle and requires Ku70/80, DNA-PKc, and DNA ligase IV [17,18,19]. HR is predominant in mid-S and mid-G2 of the cell cycle and requires the recombinase RAD51 [17,19]. Generally, there is an increased percentage of cancer cells in S and G2 phases of the cell cycle thereby favoring HR repair [20].

In prostate cancer, the AR regulates the expression of genes involved in DDR [8,9,10]. Thus, a combination of radiotherapy with ADT to block repair of DSBs leads to enhanced DNA damage and increased cell death [8,9,10]. This has led to testing combinations of radiotherapy with newer inhibitors of AR-LBD such as abiraterone, enzalutamide, apalutamide, and a novel AR-degrader, ASC-J9 [21,22,23,24,25,26]. 

One mechanism proposed to underlie poor responses to radiotherapy combined with ADT or AR-LBD inhibitors is the expression of AR-Vs. Prostate cancer cells that express AR-Vs either alone or together with full-length AR do not exhibit synergism between ADT and radiotherapy that is observed in cells that express solely full-length AR [27]. The clinical regimen of 2–3 months of neoadjuvant ADT prior to radiotherapy increases the expression of AR-Vs in prostate cancer patients [27]. These clinical data are consistent with other reports showing that ADT increases the expression of AR-Vs in prostate tissue [28,29,30]. It is suggested that in response to ADT and AR-LBD inhibitors that the concomitant upregulation of AR-Vs results in increased expression of genes that mediate DDR thereby resulting in resistance to radiotherapy [27]. A requirement for inhibition of both full-length AR and AR-Vs transcriptional activities is necessary to effectively improve cell death in response to radiotherapy [27]. 

Ralaniten and its analogs are the only known drugs proven to inhibit both full-length AR and AR-Vs by a mechanism of directly binding to AF-1 in the AR-NTD to block protein–protein interactions that are essential for its transcriptional activity [11,31,32]. Here we reveal for the first time that: (a) The next-generation ralaniten analog EPI-7170 has approximately 10 times better potency for inhibiting AR transcriptional activity compared to ralaniten; (b) ralaniten and EPI-7170 decreased mRNA and protein expression of DDR genes; and (c) a combination of ralaniten with ionizing radiation (IR) led to an accumulation of cells in S phase harboring increased DNA damage. These results reveal that blocking AR-NTD to inhibit the transcriptional activities of both full-length AR and AR-Vs combined with IR provides a new opportunity for the treatment of prostate cancer.

## 2. Results and Discussion

### 2.1. Next-Generation Ralaniten Analog, EPI-7170, Has Improved Potency against Full-Length AR and Truncated AR-Vs

**Figure 1:**
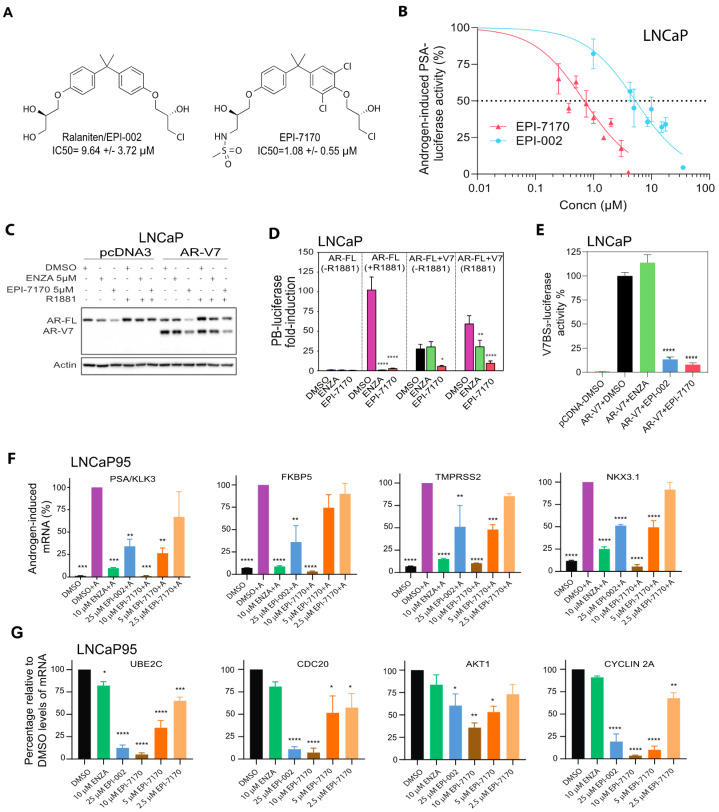
EPI-7170 is a next-generation ralaniten analog that has improved potency against the transcriptional activities of both full-length androgen receptor (AR) and active splice variants of AR (AR-Vs). (**A**) Chemical structures for ralaniten (EPI-002) and next-generation analog EPI-7170. IC50s values are for inhibition of androgen-induced PSA(6.1kb)-luciferase activity in transiently transfected LNCaP cells. (**B**) Dose-dependent inhibition of androgen-induced PSA(6.1kb)-luciferase activity in LNCaP cells. Cells were pretreated with EPI-002 and EPI-7170 before the addition of synthetic androgen R1881 (1 nM). Luciferase activities were normalized to total protein. (**C**) Levels of ectopic AR-V7 protein relative to endogenous full-length AR in LNCaP cells. Cells were transiently cotransfected with AR-V7 expression vector or empty vector (pcDNA3) and the PB-luciferase reporter and pretreated with enzalutamide (ENZA: 5 μM) or EPI-7170 (5 µM) followed by addition of R1881 (1 nM) or vehicle for an additional 24 h. Western blot analysis using antibodies to AR-NTD and β-actin with 10 μg protein of whole cells lysate. Uncut gels are provided in Figure S1. (**D**) EPI-7170 inhibits full-length AR and AR-V7 transcriptional activities. Cells were treated as in Figure 1C with or without androgen in the presence or absence of ectopic AR-V7. Data shown represent the mean ± SEM of *n* = 4 independent experiments. * *p* < 0.05, ** *p* < 0.01, **** *p* < 0.0001; Two-way ANOVA with Tukey’s multiple comparison correction. (**E**) AR-V7 transcriptional activity in LNCaP cells ectopically expressing AR-V7 and transfected with a reporter regulated by AR-V7 binding sites (V7BS_3_-luciferase). Cells were incubated with ENZA (5 µM), EPI-002 (35 µM), EPI-7170 (5 μM), or vehicle (DMSO) for 24 h. (**F**) EPI-7170 inhibits expression of endogenous genes regulated by full-length AR. LNCaP95 cells were pretreated with ENZA (10 μM), EPI-002 (25 μM), and EPI-7170 (10, 5, and 2.5 μM) prior to the addition of R1881 (1 nM), or vehicle, and incubated for an additional 48 h. Transcript levels of full-length AR target genes (PSA/KLK3, FKBP5, TMPRSS2, NKX3.1) were normalized to transcript levels of RPL13a. Bars represent the mean ± SEM of *n* = 3 independent experiments. (**G**) EPI-7170 inhibits expression of endogenous genes regulated by AR-V7. LNCaP95 cells were treated with ENZA (10 μM), EPI-002 (25 μM), and EPI-7170 (10, 5, and 2.5 μM) in the absence of androgen for 48 h. Transcript levels of AR-V7 target genes (UBE2C, CDC20, Akt1, CYCLIN 2A) were normalized to transcript levels of RPL13a housekeeping gene. Bars represent the mean ± SEM of *n* = 3 independent experiments.

Ralaniten-acetate (EPI-506) is the prodrug of ralaniten and was tested in first-in-human Phase 1 clinical trials (NCT02606123). Ralaniten-acetate is immediately converted to the active compound, ralaniten. Of the 500 ralaniten analogs that we have tested, here we describe EPI-7170 that has improved potency (Figure 1A). 

PSA(6.1kb)-luciferase and probasin (PB)-luciferase reporters are highly induced by androgen via ligand-bound full-length AR binding to androgen-response elements (AREs) within the enhancer and promoter regions of these reporters. In LNCaP human prostate cancer cells that express functional full-length AR, an IC50 for EPI-7170 was measured at 1.08 ± 0.55 μM compared to 9.64 ± 3.72 μM for ralaniten to inhibit androgen-induced PSA-luciferase activity (Figure 1B). Antiandrogens were potent as expected against androgen-induced transcriptional activity of full-length AR. Enzalutamide had an IC50 of 0.12 ± 0.04 μM; bicalutamide 0.15 ± 0.10 μM; and consistent with apalutamide not working well on the mutated T877A mutation in the AR-LBD in this cell line, it had an IC50 of 4.42 ± 1.57 μM. Ralaniten inhibits the transcriptional activities of full-length AR and truncated AR and AR-Vs lacking LBD that include AR1-653, AR-V7, and AR-V567es [11,33,34]. To ensure that the modifications to the chemical scaffold of EPI-7170 also had improved potency against the transcriptional activity of AR-V7 and in mixed populations of full-length AR and AR-Vs, robust levels of AR-V7 were ectopically expressed in LNCaP cells (Figure 1C). Equimolar concentrations of enzalutamide (5 μM) and EPI-7170 (5 μM) both effectively blocked androgen-induced transcriptional activity of full-length AR as measured using the PB-luciferase reporter (Figure 1D). Enzalutamide had no effect on AR-V7-induced transcriptional activity in the absence of androgen as expected, whereas EPI-7170 attenuated the transcriptional activity of AR-V7 (Figure 1D, middle panel). In the presence of androgen and ectopic expression of AR-V7 (Figure 1D, right panel), enzalutamide reduced PB-luciferase activity induced by full-length AR but had no effect on levels of transcription induced by AR-V7. EPI-7170 was effective at blocking the transcriptional activities of both full-length AR and AR-V7 regardless of androgen. To isolate the transcriptional activity of AR-V7, we employed the highly specific V7BS_3_-luciferase reporter that contains three AR-V7 binding sites linked in tandem in front of a minimal promoter [35]. This reporter is specific for AR-V7 with no binding sites for the full-length AR [35]. Activity of the reporter was dependent upon expression of AR-V7 and could not be blocked by enzalutamide (5 μM; Figure 1E). Both ralaniten (35 μM) and 7-fold less EPI-7170 (5 μM) were effective at blocking the transcriptional activity of AR-V7. 

To ensure that EPI-7170 blocked endogenous gene expression driven by full-length AR and AR-Vs, LNCaP95 human prostate cancer cells that express both functional full-length AR and AR-Vs were utilized [12,28,36]. Androgen-induced levels of KLK3/PSA, FKBP5, TMPRSS2, and NKX3.1 transcripts were all inhibited by enzalutamide, ralaniten, and EPI-7170 (Figure 1F). EPI-7170 was as effective or better at inhibiting androgen-induced expression of the genes compared to equimolar concentrations of enzalutamide and more than twice the concentration of ralaniten (25 μM). Androgen reduces expression of AR-Vs [37]. Therefore, to evaluate the expression of endogenous AR-V7 target genes, ADT conditions were used. Enzalutamide had little to no effect on the levels of expression of AR-V7 target genes (UBE2C, CDC20, AKT1, and cyclin 2A) whereas both ralaniten and EPI-7170 were extremely effective (Figure 1G). These data are consistent with ralaniten analogs blocking the transcriptional activity of AR-V7 with enzalutamide having no effect (Figure 1D,E) [31,33,34]. 

### 2.2. EPI-7170 Has Selectivity against AR-Dependent Proliferation

**Figure 2:**
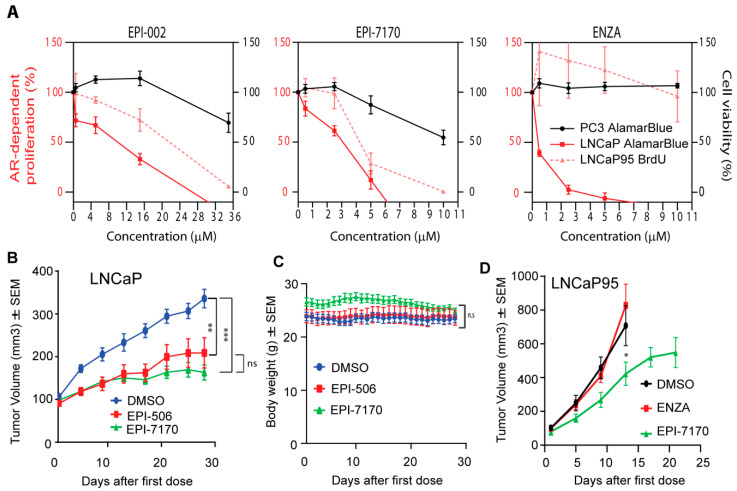
Ralaniten compounds specifically inhibit AR-dependent proliferation and tumor growth. (**A**) Androgen-induced proliferation of LNCaP cells (full-length AR), AR-V7-driven proliferation of LNCaP95 cells, and viability of AR-independent PC3 cells (lack functional AR) in response to increasing concentrations of ralaniten (EPI-002), EPI-7170 and ENZA. Proliferation of LNCaP and LNCaP95 cells was assessed by alamarBlue and BrdU incorporation, respectively, after 3 days of treatment with compounds. PC3 cells have a much faster doubling time and therefore viability (alamarBlue) was assessed after 2 days. For LNCaP cells, Y = 0 is the basal level of proliferation (DMSO in the absence of androgen) and 100% is what is achieved with androgen above that basal level. Data represent mean ± SEM of *n* = 3 independent experiments. (**B**) Ralaniten-acetate (EPI-506) and EPI-7170 inhibit LNCaP CRPC tumor growth in castrated hosts. One week after castration when tumors were approximately 100 mm^3^, animals were dosed orally daily with DMSO vehicle (3% DMSO, 1.5% Tween 80, 1% CMC), EPI-506 (22.4 mg/kg body weight) or EPI-7170 (23.3 mg/kg body weight). Mean ± SEM of *n* = 9 or 10 for each group. (**C**) Body weights of hosts were not significantly affected by treatments as described in Figure 2B. (**D**) EPI-7170 has in vivo efficacy against AR-V7-driven LNCaP95 tumors. When tumors reached 50–100 mm^3^, mice were treated for 3 weeks with DMSO vehicle, ENZA (10 mg/kg body weight) or EPI-7170 (25 mg/kg body weight) by oral gavage. Tumor volumes were measured twice a week and body weights were measured daily. Each value represents the mean ± SEM, *n* = 7. * *p* < 0.05, ** *p* < 0.01, *** *p* < 0.001; One-way ANOVA and Dunnett’s post-hoc test.

Ralaniten and its stereoisomers have specificity for AR-dependent proliferation with an IC50 in the range of 10 μM to block androgen-induced proliferation of LNCaP cells (Figure 2A, left) [11,12,31]. Clinically, ralaniten was well-tolerated at doses of up to 3.6 grams per day [14,15]. To ensure that the modifications to the chemical structure of EPI-7170 did not reduce specificity, dose–response curves were generated to compare the effects of EPI-7170 on AR-dependent proliferation versus proliferation of cells that do not depend on AR for growth and survival. EPI-7170 blocked both androgen-induced growth of LNCaP cells and androgen-independent growth of LNCaP95 cells at much lower doses than ralaniten (Figure 2A, middle). At concentrations that were at the limit of solubility (10 μM), EPI-7170 had some effect on PC3 viability. Enzalutamide was extremely potent against androgen-induced proliferation of LNCaP cells but had no effect on LNCaP95 proliferation (Figure 2A, right). These data are consistent with LNCaP95 cells being resistant to enzalutamide and their proliferation driven by AR-Vs [12,28,36].

### 2.3. In Vivo, EPI-7170 Has Antitumor Activity on CRPC Xenografts Driven by AR and AR-Vs

EPI-7170 was compared to ralaniten-acetate (EPI-506; used in Phase 1 clinical trial) for efficacy against CRPC LNCaP xenografts that express full-length AR in castrated hosts. A daily oral dose of 23.3 mg/kg body weight of EPI-7170 resulted in significant reduction of LNCaP tumor burden compared to vehicle control (Figure 2B). A daily oral dose of EPI-506 of 22.4 mg/kg body weight also reduced tumor burden significantly. Neither ralaniten nor EPI-7170 caused significant changes in body weight (Figure 2C). Importantly, EPI-7170 showed in vivo efficacy against enzalutamide-resistant LNCaP95 CRPC xenografts at an oral daily dose of 25 mg/kg body weight (Figure 2D). The growth rate of LNCaP95 xenografts was extremely aggressive in animals not treated with EPI-7170 (DMSO and ENZA) with an 8-fold increase in tumor volume in less than 2 weeks. These tumors reached maximum IRB tumor volumes in less than 3 weeks. 

### 2.4. Ralaniten and EPI-7170 Decrease Expression of DDR Genes by Targeting Both Full-Length AR and AR-Vs

**Figure 3:**
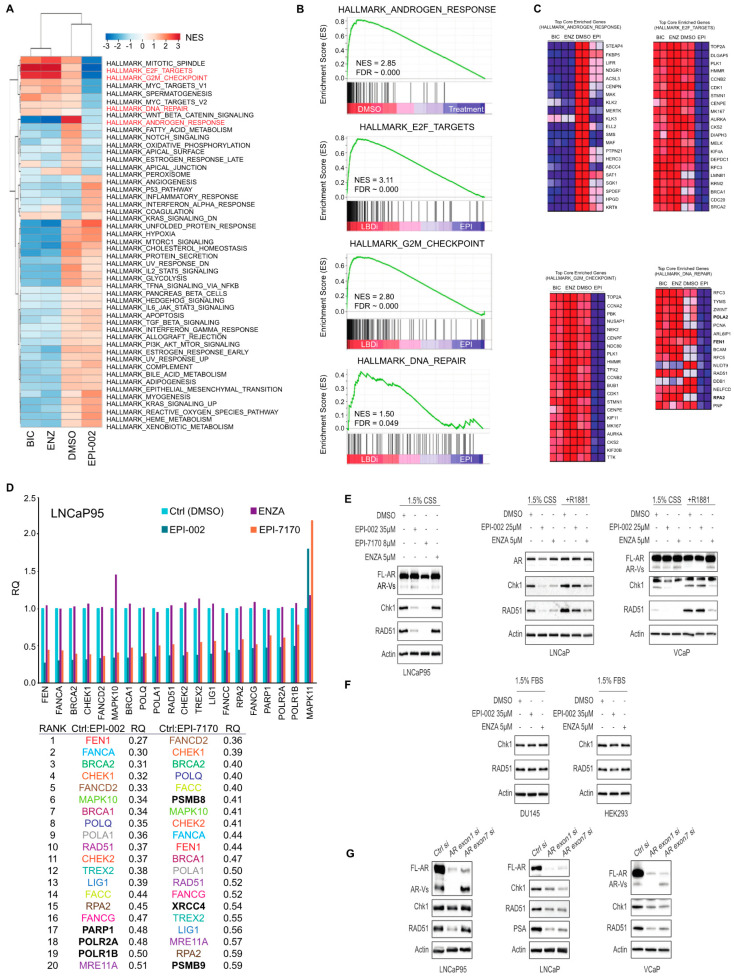
Ralaniten and EPI-7170 selectively inhibit the DNA damage repair (DDR) pathway in prostate cancer cells that express both full-length AR and AR-Vs. (**A**) Heatmap displaying normalized enrichment scores (NES) obtained from a gene set enrichment analysis (GSEA) of LNCaP cells treated with bicalutamide (BIC, 10 µM), enzalutamide (ENZA, 5 µM), ralaniten (EPI-002, 35 µM) or DMSO vehicle and stimulated with 1 nM R1881. Expression levels were normalized against control samples (DMSO/EtOH). Gene sets were restricted to MSigDB set H (hallmark gene sets). Gene sets with nominal *p* < 0.05 and false discovery rate (FDR) *q* < 0.05 were considered significant. (**B**) Enrichment plots from selected gene sets showing enriched genes in DMSO control vs. treated samples. All three AR antagonists were associated with decreased expression of genes in the HALLMARK_ANDROGEN _RESPONSE gene set (top panel). Conversely, significant enrichment was specifically seen in LBD inhibitors bicalutamide and enzalutamide compared to EPI-002 in HALLMARK_E2F_TARGETS, HALLMARK_G2M_ CHECKPOINT, and HALLMARK_DNA_REPAIR gene sets (lower three panels). (**C**) Heatmaps showing relative expression of top core enriched genes contributing to leading edge in B for each sample. (**D**) Quantitative RT-PCR to measure levels of DDR transcripts from LNCaP95 cells treated for 48 h with DMSO, EPI-002 (35 μM), ENZA (10 μM), and EPI-7170 (8 μM). RPL13a and SDHA transcript levels were applied as housekeeping genes for normalization. The top 20 genes that had altered levels of expression are listed with RQ values. The gene names in black (3 in each treatment group) were unique to that treatment. The remaining 17 were common to both analogs. Enzalutamide had no effect on the levels of expression of these genes. (**E**) Protein levels of Chk1 and RAD51 are decreased by EPI-002 and EPI-7170. Cells were incubated with compounds for 48 h and then whole cell lysates were analyzed by Western blot using antibodies to Chk1, RAD51, and then normalized to protein levels of β-actin. Uncut gels are provided in Figures S2–S4. (**F**) Ralaniten and enzalutamide have no effect on levels of Chk1 and RAD51 protein in AR-null cells (DU145 and HEK293 cells). Cells were treated as in Figure 3F. Uncut gels are provided in Figures S5 and S6. (**G**) Levels of Chk1 and RAD51 proteins are dependent on levels of full-length AR and AR-Vs. LNCaP95, LNCaP, and VCaP cells were transfected with AR siRNA targeting exon 1 or exon 7 and whole cell lysates were prepared after 48 h incubation and subjected to western blot analyses. Uncut gels are provided in Figures S7–S9.

Emerging evidence indicates that both full-length AR [7,8] and AR-Vs regulate DNA damage repair (DDR) in prostate cancer [27]. To confirm whether inhibition of AR and AR-Vs by ralaniten compounds affect the expression of DDR genes, two approaches were employed. The first was an unbiased approach in LNCaP cells to analyze the impact of two clinically relevant AR-LBD inhibitors compared to ralaniten. Enzalutamide, bicalutamide and ralaniten were effective at blocking the expression of androgen-regulated genes as expected. Importantly there were notable differences between ralaniten and LBD-inhibitors. Ralaniten decreased E2F targets, G2M Checkpoint, and importantly DNA repair (Figure 3A), whereas enzalutamide and bicalutamide either induced or had no effect on the expression of genes in these pathways (Figure 3A,B). The individual top core genes for each pathway are shown in Figure 3C. For DDR genes, ralaniten uniquely decreased expression of *POLA2, PCNA, FEN1, RAD51*, and *RPA2* to name a few in the top 15 enriched genes. To determine if ralaniten and its more potent analog EPI-7170 would block expression of DDR genes in a prostate cancer cell line that is driven by AR-Vs, a second approach employing a custom DDR qPCR array card was tested using LNCaP95 cells. Both ralaniten and EPI-7170 decreased the expression of DDR genes compared to vehicle (Figure 3D). Consistent with previous reports for the requirement to block both full-length AR and AR-Vs, enzalutamide had no inhibitory effect on the expression of genes in this pathway. Expression of the top 20 genes ranked by RQ revealed that ralaniten and EPI-7170 have similar profiles including decreased levels of expression of CHEK1 and RAD51; two well-known AR-regulated DDR genes involved in HR [8,9,38]. Protein levels of Chk1 and RAD51 were consistent with array data and support that AR and AR-Vs regulate expression of these genes. Androgen increased, whereas enzalutamide and ralaniten both decreased the expression of Chk1 and RAD51 in LNCaP and VCaP human prostate cancer cells (Figure 3E). Both ralaniten and EPI-7170 also decreased the levels of RAD51 and Chk1 in LNCaP95 cells, contrary to enzalutamide that had no effect. This is consistent with the fact that, similarly to LNCaP, VCaP cells are androgen-responsive and sensitive to AR-LBD inhibitors [39,40] while LNCaP95 cells exhibit de novo resistance to therapies targeting the AR-LBD and are instead reliant upon an AR-V7 transcriptional program [41]. In following, these data support previous reports demonstrating the ability of enzalutamide to reduce levels of Chk1 and RAD51 in androgen-dependent prostate cancer cell lines (i.e., LNCaP, LNCaP-X4-2b, and VCaP) but not in androgen-independent CWR22Rv1 cells [25,40,42]. Neither enzalutamide nor ralaniten had any impact on the levels of expression of RAD51 or Chk1 in cell lines that do not express AR as shown for DU145 and HEK293 cells (Figure 3F).

Confirmation that full-length-AR and AR-Vs regulate expression of Chk1 and RAD51 was provided by knocking down levels of full-length AR and AR-Vs using two siRNAs constructs. The first construct designed to exon 1 (encodes the AR-NTD) knocks down full-length AR and AR-Vs, whereas the second siRNA designed to exon 7 (encodes LBD) knocks down levels of full-length AR, but not AR-Vs [37,42]. Decreasing levels of full-length AR and AR-Vs by targeting exon 1 attenuated the expression of RAD51 and to some extent Chk1 in LNCaP95 cells (Figure 3G). Importantly, reduction of levels of full-length AR while maintaining levels of AR-Vs had no effect on expression of RAD51 in LNCaP95 cells (Figure 3G, left). Levels of full-length AR impacted levels of both RAD51 and Chk1 in LNCaP cells that do not express AR-Vs. For VCaP cells that express both full-length AR and AR-Vs, both AR siRNAs down-regulated the expression of Chk1 and RAD51 (Figure 3G). These are consistent with the role of full-length AR and AR-Vs to regulate expression of these DDR genes in prostate cancer cells. Together these data also support the selectivity of ralaniten and EPI-7170 to block both full-length AR and AR-Vs thereby inhibiting the expression of these DDR genes. Thus, using an inhibitor that blocks the transcriptional activities of both full-length AR and AR-Vs such as ralaniten or EPI-7170 to attenuate DDR in combination with IR therapy may yield increased cell death. 

### 2.5. Combination Therapy of Ralaniten with IR Decreases Cellular Proliferation and Survival 

**Figure 4:**
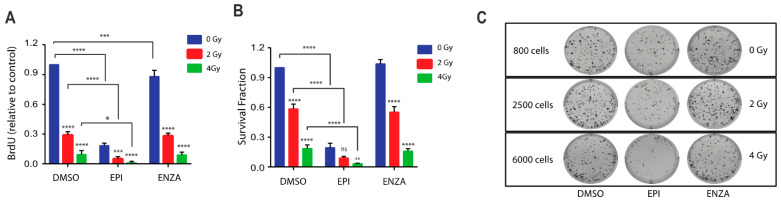
Combination therapy of ralaniten plus ionizing radiation (IR) decreases cell growth and colony formation, whereas enzalutamide has no added benefit. (**A**) LNCaP95 cells were treated with DMSO, ralaniten (EPI-002, 35 μM), ENZA (5 μM) and then 2 h later received 0, 2, and 4 Gy dose of IR. Proliferation was measured 48 h after IR using the BrdU incorporation assay. Data represent the average relative to vehicle control of *n* = 3 independent experiments ± SEM. (**B**) LNCaP95 cells were treated similarly to above and incubated for 14 days to allow colony formation. Colonies were stained with crystal violet and counted using Image J software to calculate the survival fraction. Data shown represent the mean ± SEM from *n* = 3 independent experiments. (**C**) Representative images from the colony formation assay showing the cell numbers at the start of each experiment. ** *p* < 0.01, *** *p* < 0.001, **** *p* < 0.0001; ns, not significant. Two-way ANOVA with Tukey’s multiple comparison test was used.

Blocking full-length AR transcriptional activity enhances DNA damage in response to radiotherapy [7,8,9,22] but unfortunately the expression of AR-Vs circumvent this blockage thereby leading to the requirement for blocking both full-length AR and AR-Vs [27]. Thus, we hypothesized that a combination of ralaniten with IR should result in increased DNA damage and reduced proliferation and survival. To test this, LNCaP95 cells were treated with ralaniten or enzalutamide for two hours prior to IR. Ralaniten monotherapy markedly reduced proliferation while enzalutamide monotherapy had less of an effect (Figure 4A). A combination of IR (2 Gy or 4 Gy) with ralaniten yielded significantly better responses compared to monotherapies (Figure 4A). A combination of enzalutamide with IR did not improve responses compared to IR monotherapy thereby the addition of enzalutamide had no benefit. A similar trend was measured using the clonogenic assay that revealed improved responses with a combination of ralaniten and IR and no benefit by adding enzalutamide with IR (Figure 4B,C). The combination of IR with ralaniten was additive in BrdU assays measuring proliferation (Combination Index for 2Gy = 1.004, and for 4 Gy = 1.008) and also with the clonogenic assay (Combination Index for 2Gy = 1.025, and for 4 Gy = 1.004).

### 2.6. Combination Therapy of Ralaniten with IR Induces Cell Cycle Arrest in S-Phase 

**Figure 5:**
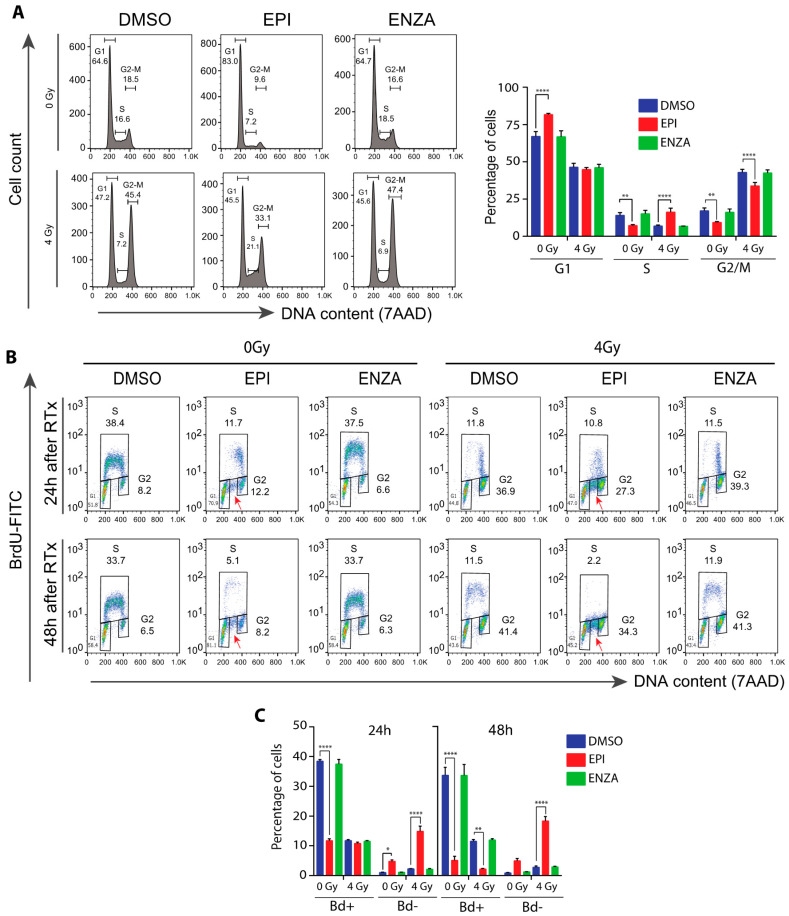
Combination therapy of IR plus ralaniten maximizes blockade of DNA synthesis and increases accumulation of cells stalled in S phase of the cell cycle. EPI alone caused G1 arrest. IR alone caused G2/M arrest. EPI plus IR caused greater S arrest so that cells were not able to continue to G2/M and then G1. LNCaP95 cells were pretreated for 2 h with DMSO, ralaniten (EPI; 35 μM), ENZA (5 μM) prior to IR and then incubated an additional 24 or 48 h. After labelling for 2 h with 10 µM BrdU, the cells were stained with BrdU-FITC antibody and 7-AAD, and subjected to FACS analysis. (**A**) Representative histograms of 7-AAD stained cells 48 h after IR are shown (left panel) and the average percentages of cells in each phase of the cell cycle (right panel). (**B**) Representative bivariate plots of BrdU and 7-AAD for cells exposed to monotherapy or combinations for 24 or 48 h, as indicated above. The fraction of cells in G1, S, and G2 phases are indicated next to the corresponding box. The red arrows designate cells in S phase that did not incorporate BrdU. (**C**) Quantification of BrdU-positive (gated as S) and BrdU-negative (indicated by red arrows) cells from bivariate plots after 24 or 48 h of treatments. For A and C, the bars represent the means ± SEM from *n* = 3 independent experiments. * *p* < 0.05, ** *p* < 0.01, **** *p* < 0.0001; ANOVA with Tukey’s multiple comparisons test was used.

DDR is dependent on the cell cycle phase and checkpoints. Consistent with blocking AR transcriptional activity, ralaniten induces LNCaP cells to arrest in G1 similar to ADT and AR-LBD inhibitors [31,34]. A functional AR is required for entry of G1 cells into S phase [43,44]. LNCaP95 cells are androgen-independent with their proliferation dependent upon AR-Vs [12,28,36]. Thus, as expected, enzalutamide had no effect on cell cycle progression relative to the control (Figure 5A). Ralaniten induced G1 cell cycle arrest as expected for an inhibitor of AR-Vs. IR induced a 2- to 3-fold increase of cells in G2/M compared to non-IR treated cells. However, this effect was attenuated by pretreatment with ralaniten, but not with enzalutamide. In addition, the combination of ralaniten with IR increased the number of cells in S-phase (Figure 5A). 

Interestingly, ralaniten caused a 3-fold reduction in the percentage of cells incorporating BrdU compared to the DMSO control after 24 h and this reduction was mainly in early S-phase cells (Figure 5B, left side of the subpopulation in BrdU-positive cells). Moreover, G1 cells increased from 24 h to 48 h of ralaniten treatment. These data suggest that the cells in late S-phase (i.e., right side sub-population in BrdU-positive cells) observed after 24 h of treatment with ralaniten had progressed in the cell cycle and were arrested in G1 by 48 h of treatment. Notably, ralaniten treatment caused an accumulation of BrdU-negative cells in S phase (Figure 5B, red arrows), indicating a portion of S cells that were not able to incorporate BrdU. Similar to ralaniten, IR alone reduced cells in early S-phase. However, IR alone did not cause accumulation of BrdU-negative cells in S phase. Furthermore, the percentage of BrdU-negative cells was significantly higher in cells treated with ralaniten plus IR compared with ralaniten alone or IR alone (Figure 5C). This result suggests the inability of these cells to progress to G2/M and thereby they remain arrested in S phase. This intra-S phase arrest in response to ralaniten could result from DNA damage or DNA replication stress, and was exacerbated with radiation. Together these data suggest that the combination of ralaniten with radiation leads to increased cell cycle arrest, particularly in S phase.

### 2.7. Combination of Ralaniten and IR Induces Accumulation of DSBs 

**Figure 6:**
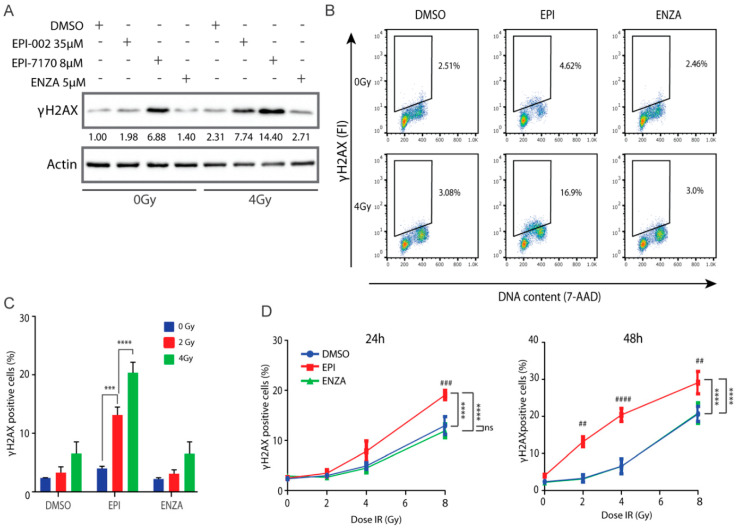
Ralaniten and EPI-7170 increase levels of γH2AX whereas enzalutamide has little added benefit. (**A**) LNCaP95 cells were pretreated for 2h with DMSO, ralaniten (EPI-002), EPI-7170, and ENZA prior to IR (0 or 4 Gy) and then incubated for an additional 48 h before whole cell lysates were analyzed by Western blot using antibodies to γH2AX and β-actin. Fold-induction values are provided under each γH2AX protein band which were normalized to β-actin and basal levels in non-treated cells (DMSO, column 1). Uncut gels are provided in Figure S10. (**B**) Representative bivariate plots of cells stained with γH2AX antibody and 7-AAD at 48 h after IR and pretreatment with DMSO, EPI-002 (EPI; 35 μM), or ENZA (5 μM). (**C**) LNCaP95 cells were treated as in B, stained for γH2AX and analyzed by Flow Cytometry to measure the percentage of γH2AX-positive cells. (**D**) Percentage of γH2AX positive cells at 24 h and 48 h after various doses of IR and 2h pretreatment with DMSO, EPI-002 (EPI; 35 μM) or ENZA (5 μM). Bars and dose–response curves represent the mean ± SEM of *n* = 3 independent experiments. Ns, not significant. *** *p* < 0.001, **** *p* < 0.0001; ANOVA test with Tukey’s test. Comparison between DMSO (vehicle) and EPI-002 at each IR dose: ##, *p* < 0.01; ###, *p* < 0.001; ####, *p* < 0.0001.

γH2AX is a marker for DNA damage [45,46,47,48,49] and aids in prediction of cell death [50,51,52,53]. To determine if combining ralaniten or EPI-7170 with IR could increase the induction or delay the repair of DSBs in prostate cancer cells that express both full-length AR and AR-Vs, levels of γH2AX were analyzed by Western blot of LNCaP95 cell lysates. Higher levels of γH2AX were detected in cells treated for 48 h with ralaniten and EPI-7170 compared to levels in the control and enzalutamide-treated cells (Figure 6A). IR increased levels of γH2AX with maximum levels observed with the combination with ralaniten or EPI-7170, compared to the levels of γH2AX in DMSO vehicle and enzalutamide-treated cells (Figure 6A). Analysis of γH2AX expression using flow cytometry with the fluorescence intensity normalized to DNA content to correct for DNA differences during the phases of the cell cycle [54] was consistent with western blot analyses. In the absence of IR, there was approximately a two-fold increase in the percentage of cells retaining residual γH2AX when treated with ralaniten compared to the vehicle (Figure 6B). Cells treated with a combination of enzalutamide and IR had resolved DNA damage that was similar to the vehicle control (Figure 6B,C). Analysis of residual γH2AX with increasing amounts of IR revealed a higher proportion of cells with residual γH2AX in those pretreated with ralaniten in relation to vehicle control or enzalutamide, this effect was both dose- and time-dependent (Figure 6D). Together these data support the application of IR with ralaniten for the treatment of prostate cancer that expresses full-length AR and AR-Vs.

## 3. Materials and Methods

### 3.1. Cells and Inhibitors 

Cell lines were obtained from the following: LNCaP cells from Dr. Leland Chung (Cedars-Sinai Medical Center, Los Angeles, CA, USA) in September 1993, LNCaP95 from Stephen R. Plymate (University of Washington, Seattle, WA, USA), PC3, VCaP and HEK293 were from ATCC (Manassas, VA, USA), DU145 from Victor Ling (British Columbia Cancer Agency, Integrative Oncology) in October 1998. Each cell line was maintained in RPMI-1640 (for LNCaP, LNCaP95, C4-2B), MEM (for HEK293), or DMEM (PC3, VCaP and DU145) medium supplemented with 5% or 10% FBS or charcoal-stripped serum (CSS). Enzalutamide was purchased from Omega Chem (Saint-Romuald, QC, Canada). Bicalutamide from a gift from Dr. Marc Zarenda (AstraZeneca, Cambridge, England). Ralaniten and EPI-7170 were synthesized by us.

### 3.2. Reporter Assays

PSA(6.1kb)-luciferase, PB-luciferase, V7BS_3_-luciferase and AR-V7 plasmids and transfection protocols for LNCaP cells have been described [11,32,36]. IC50 values were calculated using GraphPad Prism 7 (GraphPad Software, Inc., La Jolla, CA, USA).

### 3.3. Cell Viability and Proliferation Assay

LNCaP cells (5000 cells/well) and PC3 cells (2000 cells/well) were plated in 96-well plates in their respective media plus 1.5% CSS. The next day, PC3 cells were treated with compounds for 2 days. LNCaP cells were pretreated with compounds before incubating with 0.1 nM R1881 for 3 days. Proliferation and viability were measured using AlamarBlue cell viability assay (ThermoFisher Scientific, Waltham, MA, USA) following the manufacturer’s protocol. LNCaP95 cells (6000 cells/well) were seeded in 96-well plates in RPMI media supplemented with 1.5% CSS for 48 h. Cells were treated with compounds for 48 h. BrdU incorporation was measured after 2 days using BrdU Elisa kit (Roche Diagnostics, Laval, QC, Canada).

### 3.4. Gene Expression Analysis

LNCaP95 cells (180,000 cells/well) were plated in 6-well plates. The cells were treated with vehicle, ENZA, and EPI-7170 for one hour prior to treating with, or without 1 nM R1881 for 48 h. Total RNA was isolated using pure link RNA isolation kit (ThermoFisher Scientific) and reverse transcribed with high capacity RNA to cDNA kit (ThermoFisher Scientific). Quantitative real-time PCR was performed using *n* = 3 independent experiments with technical triplicates for each sample. Expression levels were normalized to levels of RPL13A transcript. Primers have been described [11]. 

### 3.5. Microarray and GSEA Analysis

Total RNA was extracted from LNCaP cells treated with ralaniten (35 µM), enzalutamide (5 µM), bicalutamide (10 μM), or vehicle (DMSO) and stimulated with either 1 nM R1881 or ethanol (EtOH) vehicle. RNA was reverse transcribed and cDNA was hybridized to the GeneChip Human Transcriptome Array 2.0 from Affymetrix. RT-PCR, cDNA hybridization and chip reading were carried out at CDRD’s Target Validation Division at the University of British Columbia (Vancouver, BC, Canada; www.cdrd.ca). Analysis of raw signal output was done using GeneSpring software (version 13.1). Hierarchical clustering was performed by conducting a 2-Way ANOVA on data with a significance threshold set at 0.05, and expression was normalized to DMSO/EtOH control samples. The Benjamini–Hochberg correction was applied to reduce the false discovery rate (FDR).

Data generated from the microarray analysis was used to identify differences between AR antagonists. GSEA version 7.0 software (http://software.broadinstitute.org/gsea/msigdb/index.jsp) was used, and the difference in the expression levels between vehicle and drug treatment for each gene was analyzed based on the Molecular Signatures Database set H (Hallmark gene sets, h.all.v7.1.symbols.gmt). The permutation number was set to 1,000. Those enrichment gene sets revealed by GSEA as exhibiting a nominal *p* < 0.05 and *FDR* < 0.05 were considered to indicate a statistically significant difference. The default parameters were used in GSEA software. Hierarchical clustering was performed on normalized enrichment scores (NES) generated for each sample using ClustVis software (https://biit.cs.ut.ee/clustvis/#general).

### 3.6. TaqMan™ Custom Gene Expression Array Fast Plates

A custom Taq Man 96-well Fast Gene expression plate (Thermo Fisher Scientific) was used according to the manufacturer’s protocol using StepOne Plus Real-Time PCR System. Data were analyzed using ExpressionSuite Software Version 1.1 (Thermo Fisher). Quant Studio 6 Flex Real-Time PCR System (Applied Biosystems, Beverly, MA, USA) and Platinum SYBR Green qPCR SuperMix (Thermo Fisher Scientific) were used for conventional qPCR. Expression levels were normalized to levels of RPL13a transcript. 

### 3.7. SiRNA Transfection

siRNA targeting AR exon 1 and exon 7 were designed according to previous reports [38,55] and purchased from Dharmacon (Lafayette, CO, USA). The target sequence for AR exon 1 and exon 7 were [5’-CAAGGGAGGUUACACCAAAUU-3’] and [5’-GGAACUCGAUCGUAUCAUU-3’] respectively. siRNA knockdown was performed using Lipofectamine RNAiMAX (Invitrogen, Carlsbad, CA, USA) according to the manufacturer’s protocol [MAN0007825 Rev.1.0].

### 3.8. IR and BrdU Incorporation

LNCaP95 cells were pretreated for two hours with compounds prior to IR with doses of 2 or 4 Gy using a Precision X-Ray XRAD320 (300 KeV and 10 mA with 1.5 mm Al, 0.25 mm Cu and 0.75 mm Sn beam conditioning filter at room temperature). BrdU incorporation was measured 48 h after IR, using the Cell Proliferation ELISA BrdU kit (Roche, Basel, Switzerland).

### 3.9. Clonogenic Assay

LNCaP95 cells were treated with DMSO vehicle, ralaniten, or ENZA in 6-well plates. Two hours later, cells were irradiated with doses of 0, 2, or 4 Gy and incubated for an additional 14 days. Colonies were fixed with 4% paraformaldehyde, stained with 0.1% crystal violet for 20 min and then photographed using ChemiDoc (Bio-Rad, Hercules, CA, USA). Colonies containing more than 50 cells were counted using ImageJ software ver. 1.45s (Rasband, W.S., ImageJ, U.S. National Institutes of Health, Bethesda, MD, USA).

### 3.10. Western Blots and Antibodies

Cells were seeded in 6-well plates and treated 24 or 48 h (LNCaP95) later with DMSO, ralaniten, EPI-7170 or ENZA in media supplemented with 1.5% CSS. Cells were harvested 24 h later and 20 µg of total protein was separated on Tris-Glycine gels and transferred to PVDF membrane (0.45 µm PVDF from Millipore and 0.2 µm PVDF from Bio-Rad). The membranes were immunoblotted with the following antibodies and dilutions (1:1000) AR N-20 (SC-816), Chk1 (G-4, SC-8408) from Santa Cruz Biotechnology (Dallas, TX, USA); RAD51 (D4B10, #8875), γH2AX (Ser139, #9718) and PSA (#2475) from Cell Signalling Technology (Beverly, MA, USA), and β-actin (1:10000, Abcam, Cambridge, MA, USA). After overnight incubation with primary antibodies, the membranes were incubated with horseradish peroxidase-conjugated secondary antibodies. Western blots were developed with Amersham ECL or ECL Prime Western Blotting Detection Reagent (GE Healthcare Life Sciences, Marlborough, MA, USA). Membranes were stripped in Restore™ Western Blot Stripping Buffer (Thermo Fisher Scientific) for re-probing. ImageJ was used for the densitometry analyses.

### 3.11. FACS Analysis

LNCaP95 cells seeded into 10 cm dishes in 1.5% CSS were pretreated with DMSO, ralaniten, or ENZA. Two hours later, cells received IR doses of 0 or 4 Gy and then were incubated for an additional 48 h. Cells were labelled with BrdU for 2 h before being harvested and fixed in 70% ethanol. For cell cycle analysis, cells were incubated with BrdU-FITC antibody (BD Biosciences, Franklin Lakes, NJ, USA). DNA was stained with 7-aminoactinomycin D (7-AAD, Sigma-Aldrich, Saint Louis, MO, USA). For γH2AX analysis, cells were incubated first with γH2AX antibody (Ser139, #9718, 1:500) followed by AlexaFluor 488 anti-rabbit (1:200). DNA was stained with 7-AAD. Data were acquired using a FACS Calibur (BD Biosciences) and analyzed using FlowJo software ver.10.3 (Ashland, OR, USA).

### 3.12. Animal Xenograft Model 

All animal experiments were approved by the University of British Columbia Animal Care Committee. For the LNCaP xenograft model, six- to eight-weeks old male NOD-SCID gamma mice were inoculated subcutaneously with 5 × 10^6^ cells/tumor in both flanks. When the tumor volume reached 50–100 mm^3^, mice were castrated and 7 days later they received daily oral doses of DMSO, EPI-506 (66 mg/kg, s.i.d.) or EPI-7170 (25 mg/kg, s.i.d.). For the LNCaP95 xenograft model, NOD-SCID gamma mice were castrated two weeks before subcutaneous inoculation with 2 × 10^6^ cells/tumor in both flanks. When the tumor volume reached 50–100 mm^3^, the animals received daily oral doses of DMSO, EPI-7170 (25 mg/kg, s.i.d.) or ENZA (10 mg/kg, s.i.d.). Body weight was measured every day and tumor volume was measured twice a week using a caliper. Tumor volume was calculated by the formula: length × width × height × 0.5236. 

### 3.13. Statistical Analysis

The data reported are shown as the mean ± SEM. Unless stated otherwise, statistical significance was determined by ANOVA using Dunnett’s or Tukey’s multiple comparisons test. An alpha level of 0.05 was used for all statistical tests, which is summarized in the figures as * *p* < 0.05, ** *p* < 0.01, *** *p* < 0.001, **** *p* < 0.0001; ns, not significant.

## 4. Conclusions

In summary, these data reveal that blocking the transcriptional activities of both full-length AR and truncated AR-Vs with ralaniten inhibits the expression of DDR genes, cell proliferation, as well as cell survival, thereby leading to increased sensitivity of prostate cancer to radiotherapy. Clinical applications include localized prostate cancer (combined with possibly external beam radiotherapy or brachytherapy) as well as for advanced disease including CRPC, oligoprogressive CRPC, and oligometastatic prostate cancer by combining ralaniten analogs with stereotactic radiation therapy, radium-223, or targeted alpha emitters such as 225Ac-labeled PSMA-617.

## Supplementary Materials

The following are available online at https://www.mdpi.com/2072-6694/12/7/1991/s1, Figures S1–S10: The whole western blots.
