# Supplementary material for: Ralaniten Sensitizes Enzalutamide-Resistant Prostate Cancer to Ionizing Radiation in Prostate Cancer Cells that Express Androgen Receptor Splice Variants
Source: Cancers (Basel). 2020 Jul 21;12(7):1991. doi: 10.3390/cancers12071991 (PMC7409302; doi:10.3390/cancers12071991)
Supplement: Supplementary file 1 [file cancers-12-01991-s001.pdf]

Supplementary Figure S1
(related to Figure 1C LNCaP)

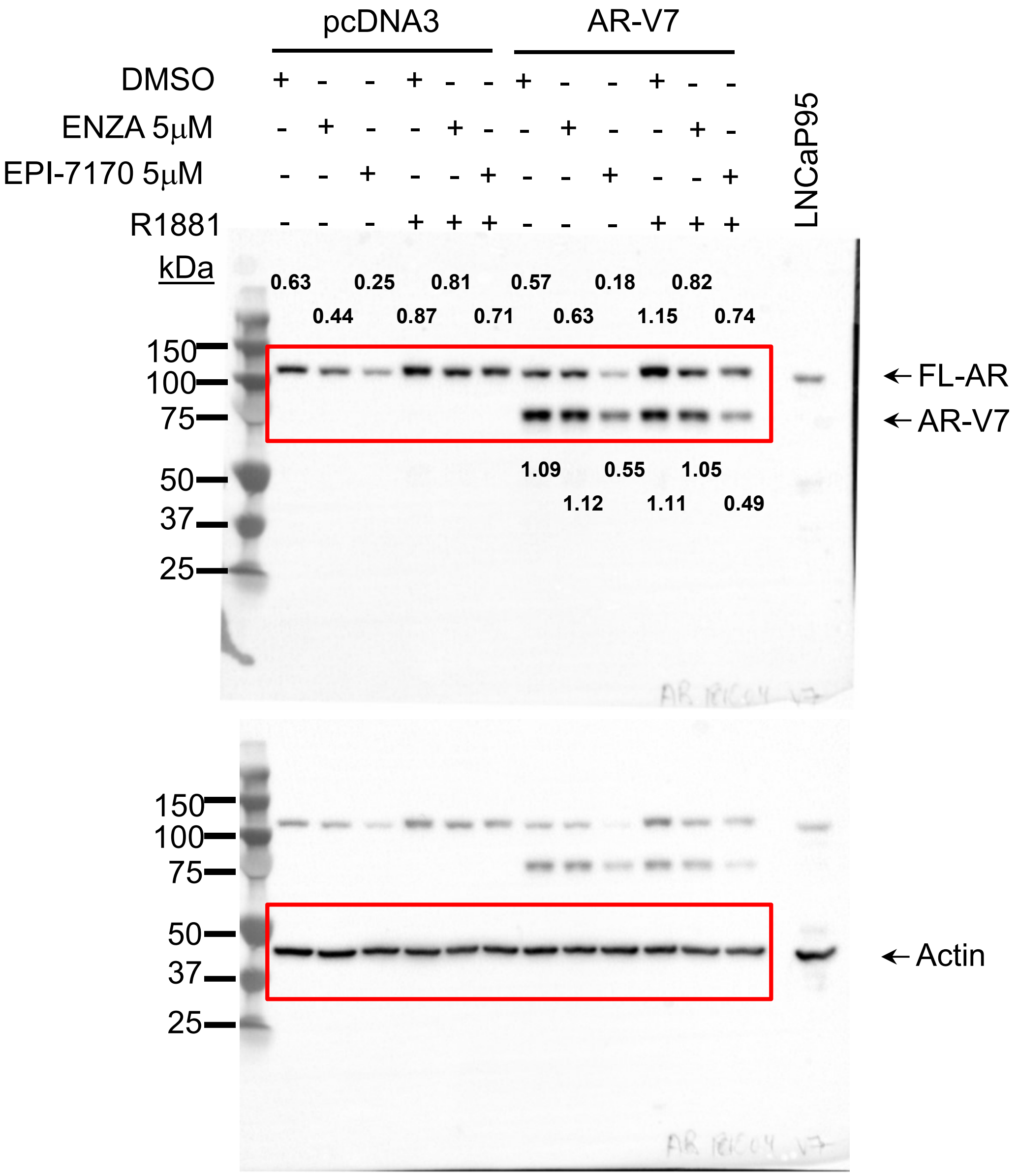

Supplementary Figure S1 (related to Figure 1C LNCaP)
Whole Western blots are shown. Intensity of each band is normalized to that of actin and ratios are indicated on the figure. Red rectangles indicate the proteins of interest.

Supplementary Figure S2
(related to Figure 3E LNCaP95)

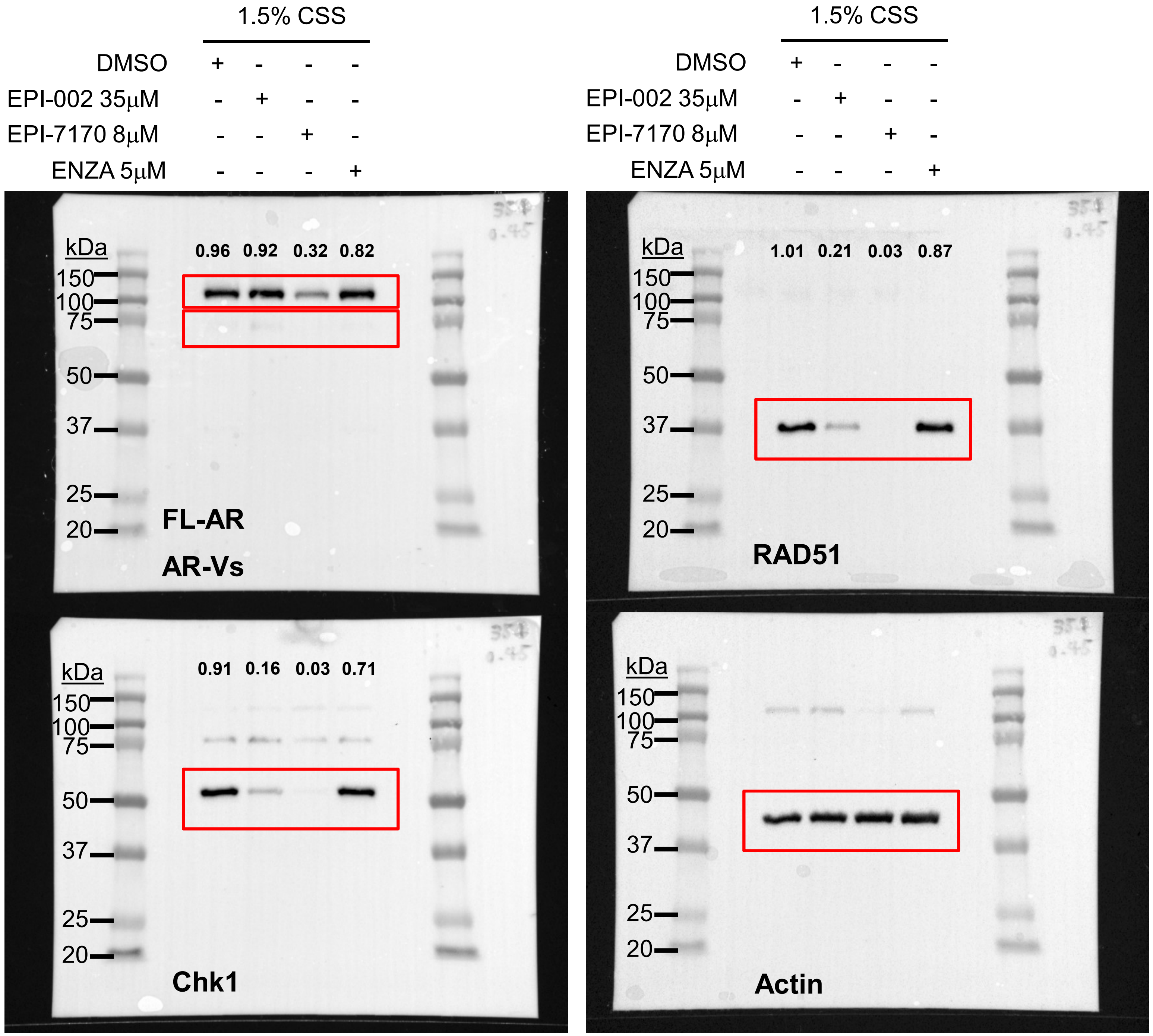

Supplementary Figure S2 (related to Figure 3E LNCaP95)
Whole Western blots are shown. Intensity of each band is normalized to that of actin and ratios are indicated on the figure. Red rectangles indicate the proteins of interest.

Supplementary Figure S3
(related to Figure 3E LNCaP)

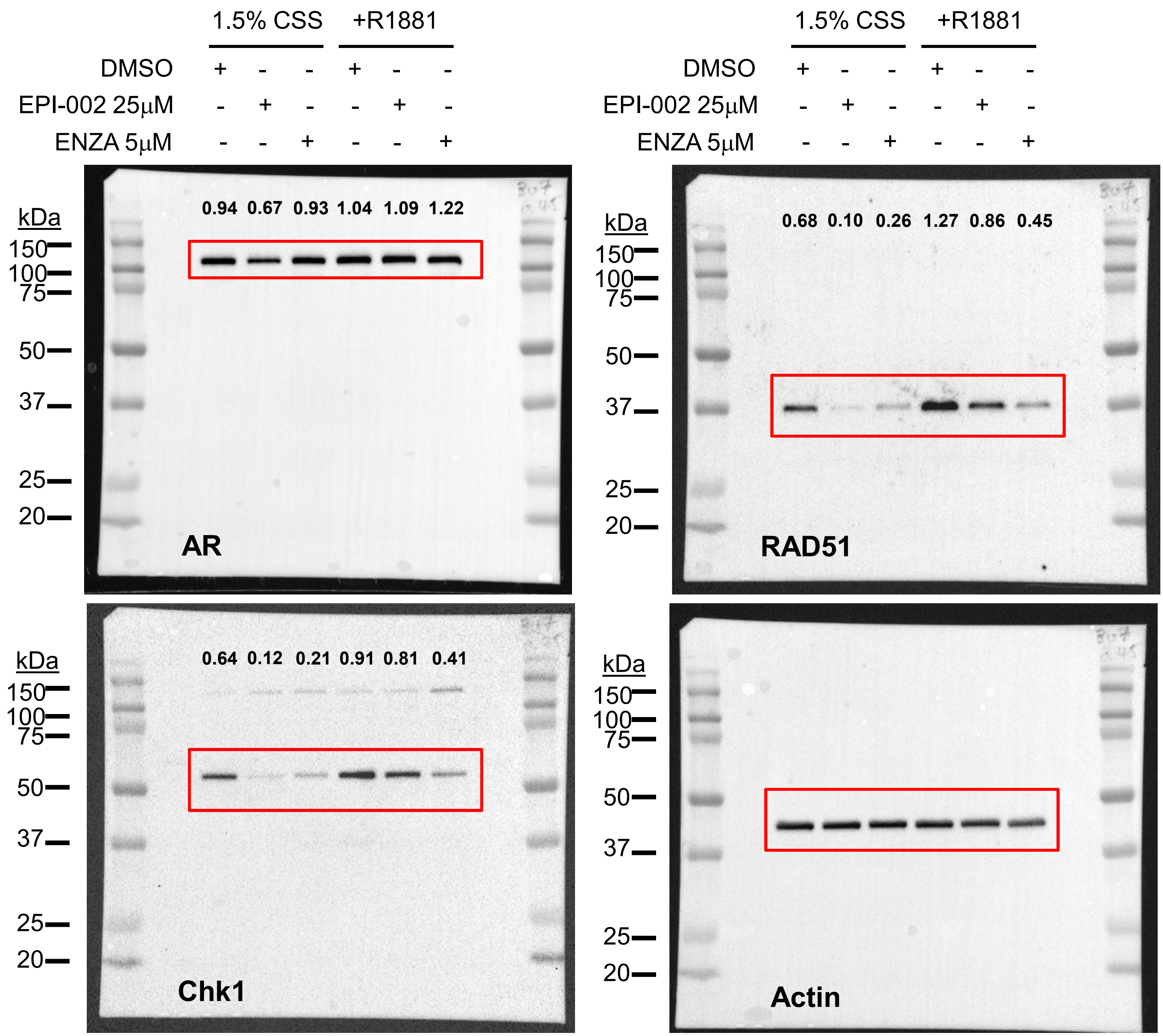

Supplementary Figure S3 (related to Figure 3E LNCaP)
Whole Western blots are shown. Intensity of each band is normalized to that of actin and ratios are indicated on the figure. Red rectangles indicate the proteins of interest.

Supplementary Figure S4
(related to Figure 3E VCaP)

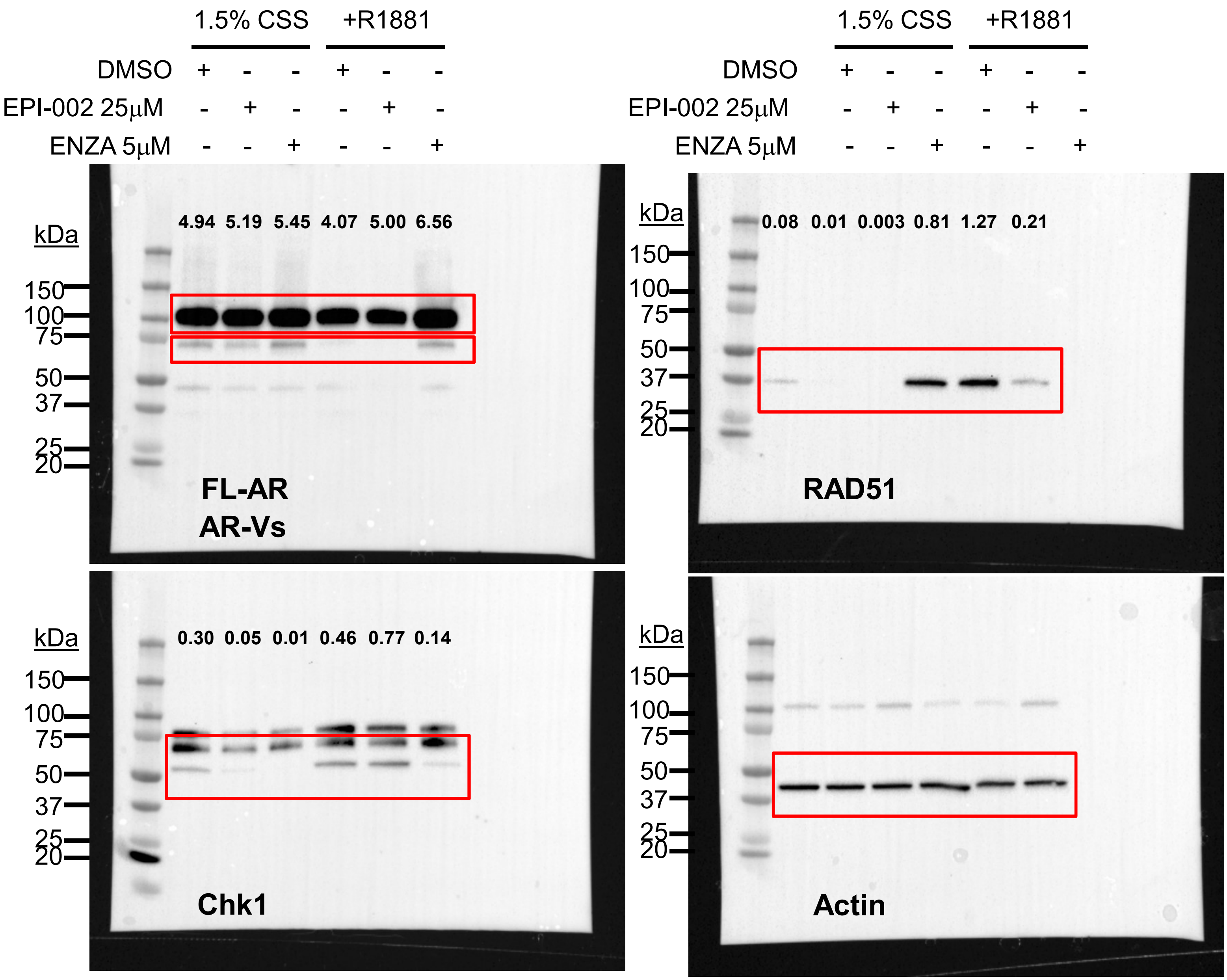

Supplementary Figure S4 (related to Figure 3E VCaP)
Whole Western blots are shown. Intensity of each band is normalized to that of actin and ratios are indicated on the figure. Red rectangles indicate the proteins of interest.

Supplementary Figure S5
(related to Figure 3F DU145)

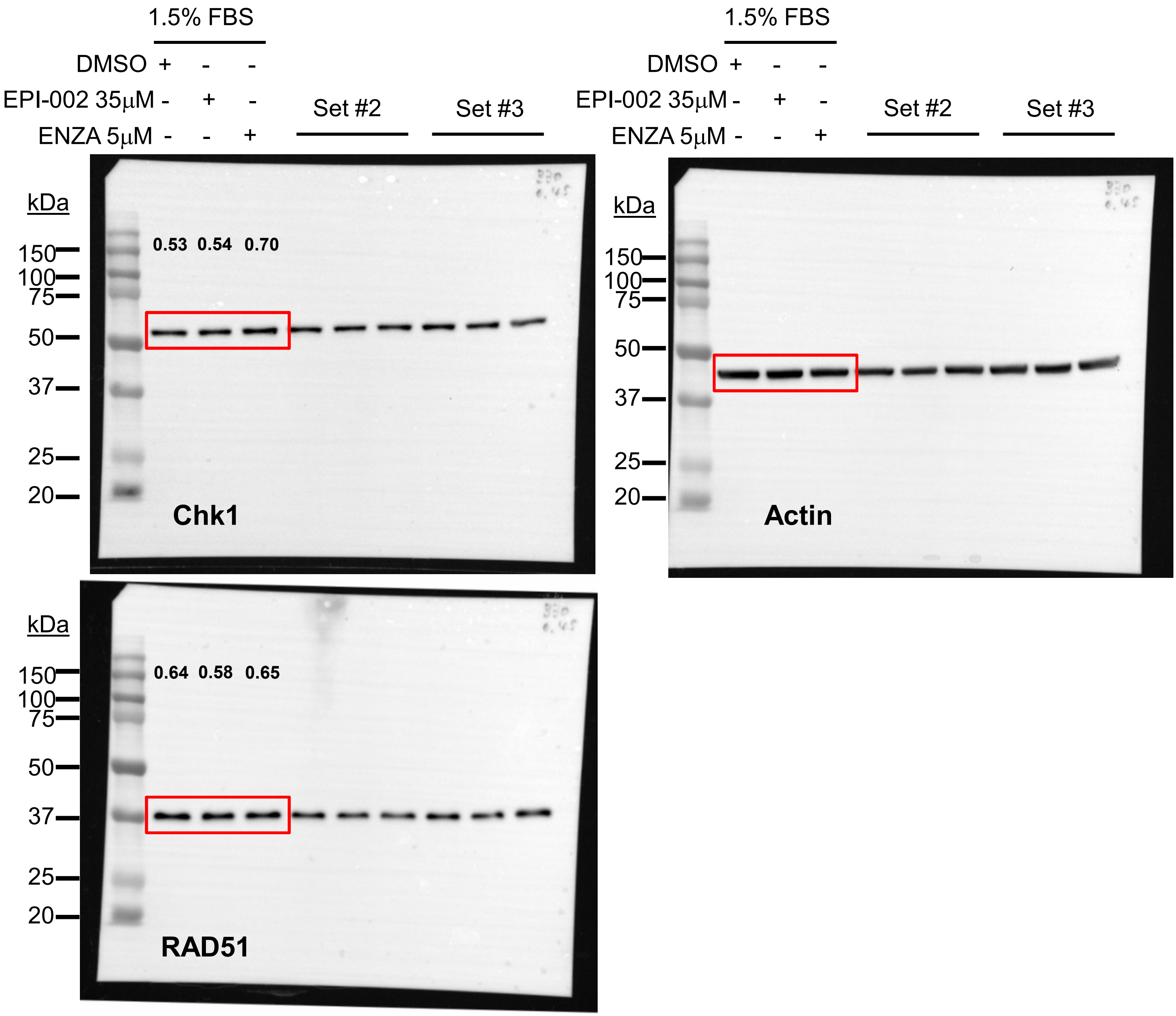

Supplementary Figure S5 (related to Figure 3F DU145)
Whole Western blots are shown. Intensity of each band is normalized to that of actin and ratios are indicated on the figure. Red rectangles indicate the proteins of interest.

Supplementary Figure S6
(related to Figure 3F HEK293)

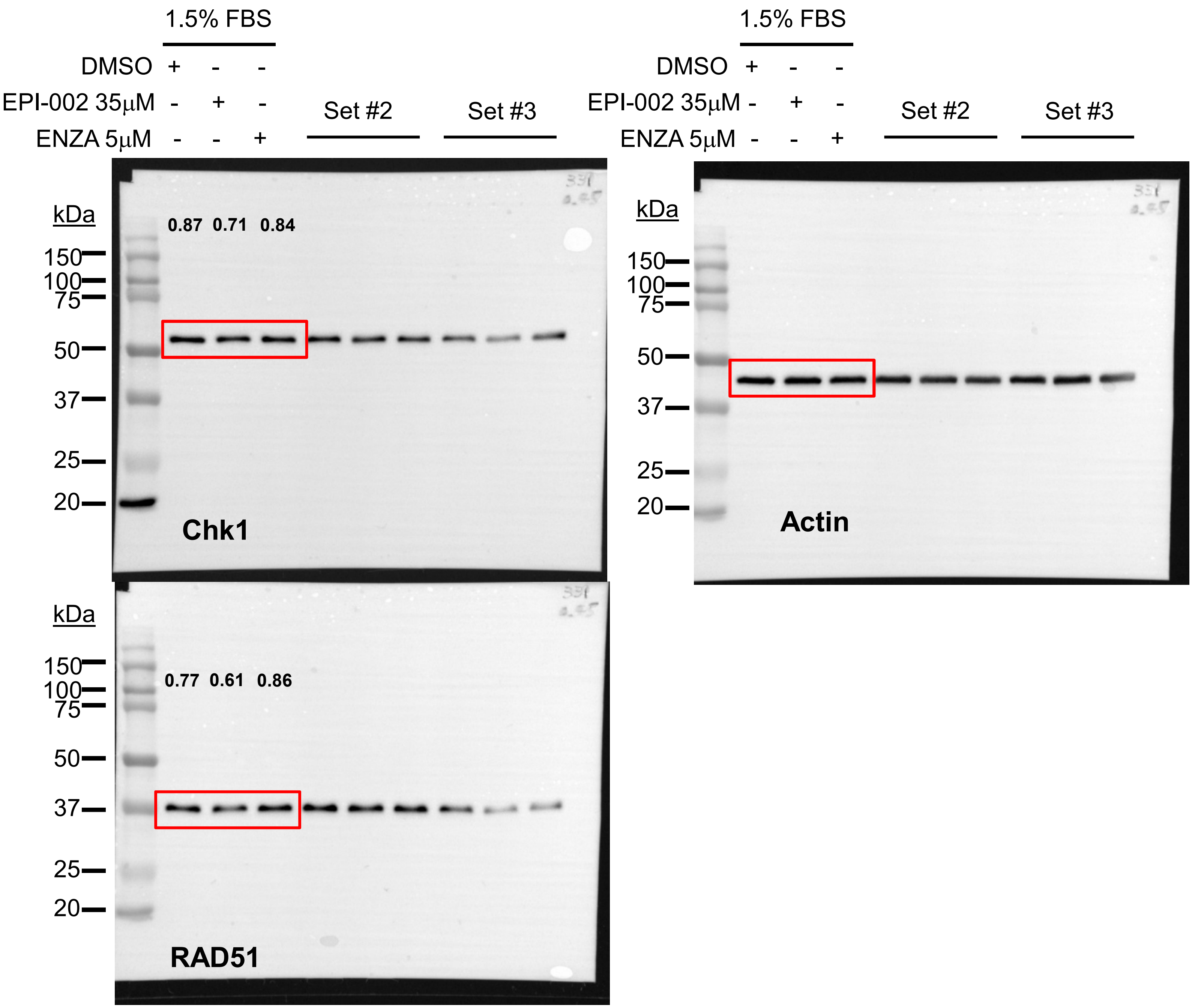

Supplementary Figure S6 (related to Figure 3F HEK293)
Whole Western blots are shown. Intensity of each band is normalized to that of actin and ratios are indicated on the figure. Red rectangles indicate the proteins of interest.

Supplementary Figure S7
(related to Figure 3G LNCaP95)

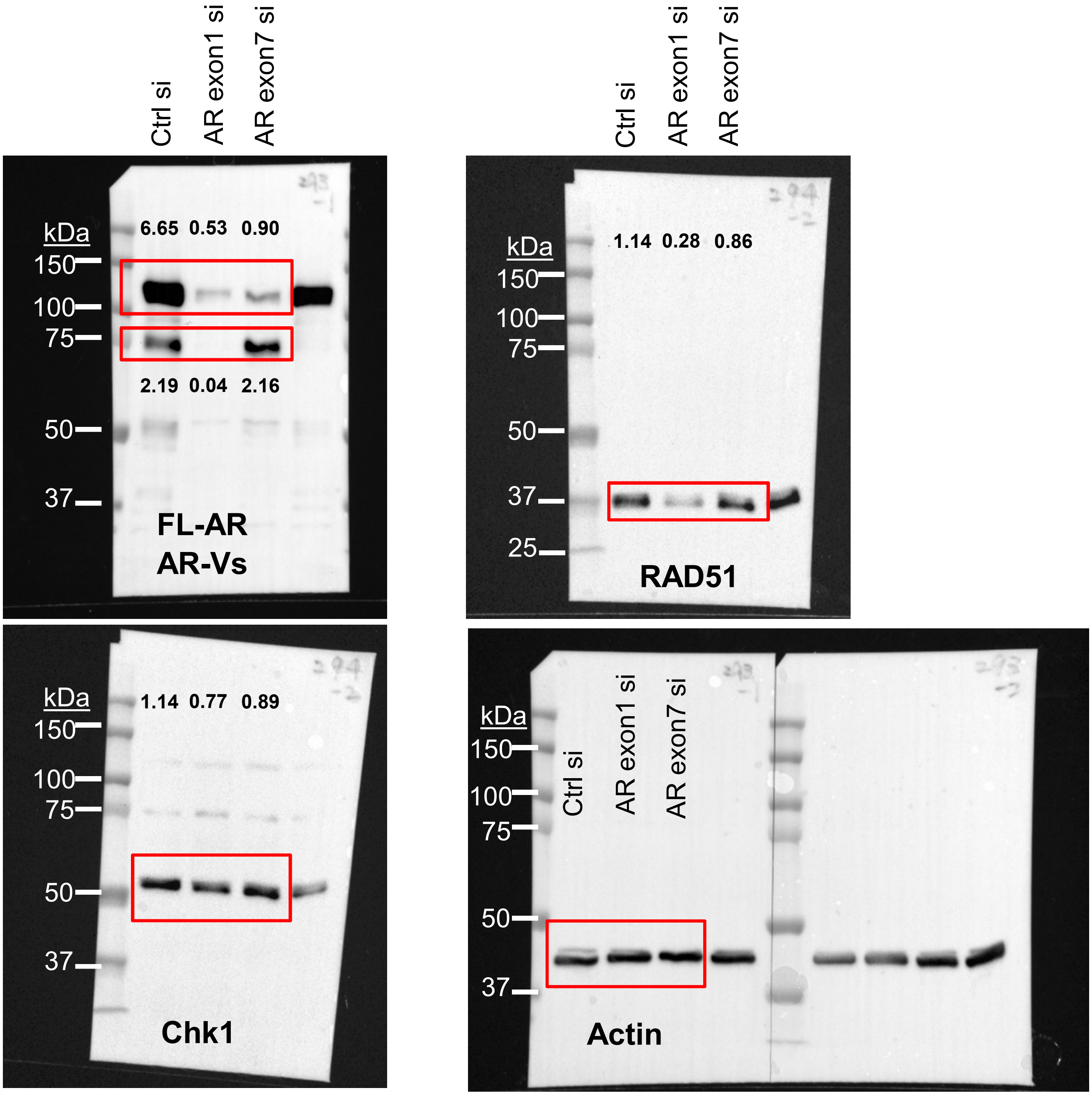

Supplementary Figure S7 (related to Figure 3G LNCaP95)
Whole Western blots are shown. Intensity of each band is normalized to that of actin and ratios are indicated on the figure. Red rectangles indicate the proteins of interest.

Supplementary Figure S8
(related to Figure 3G LNCaP)

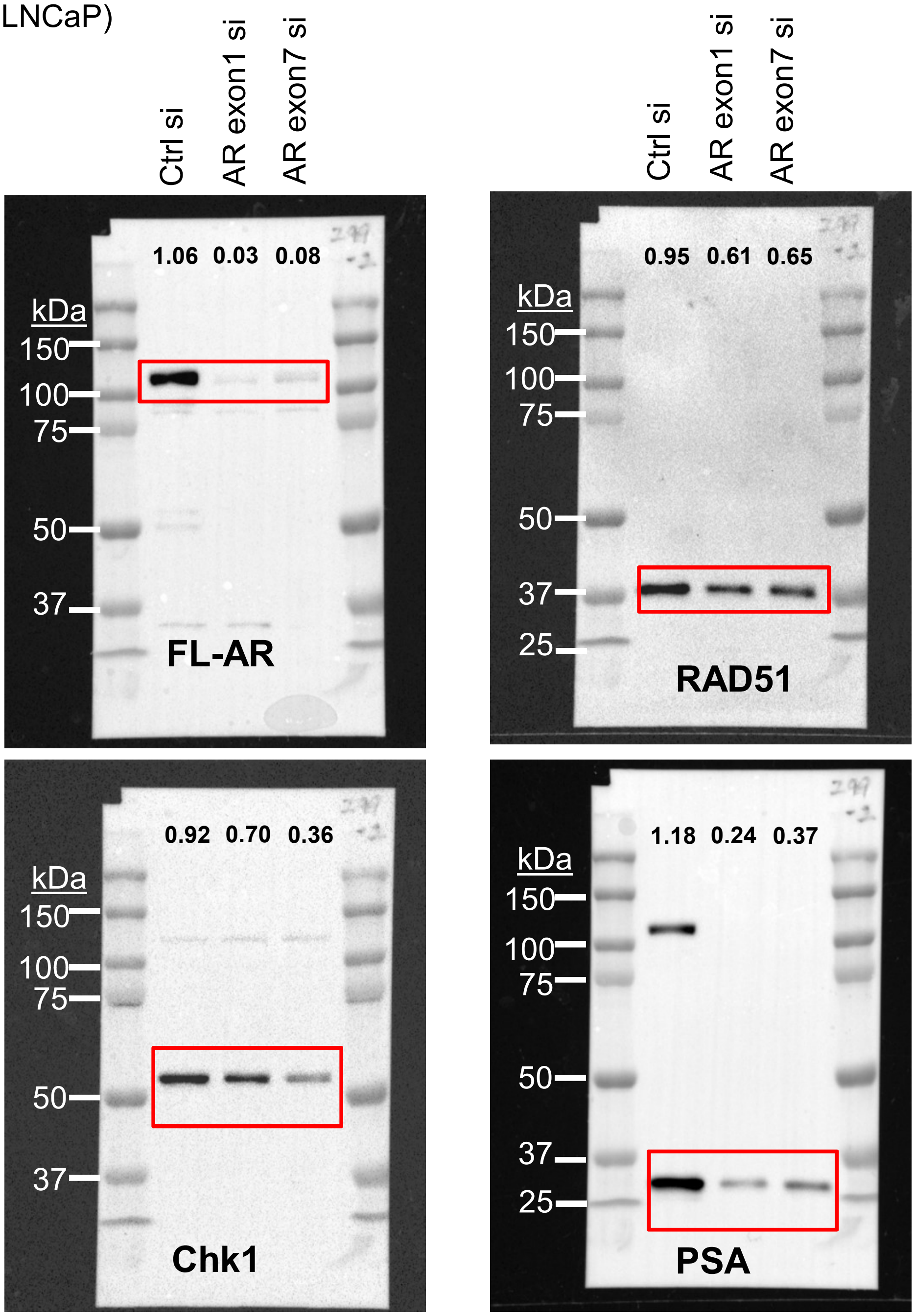

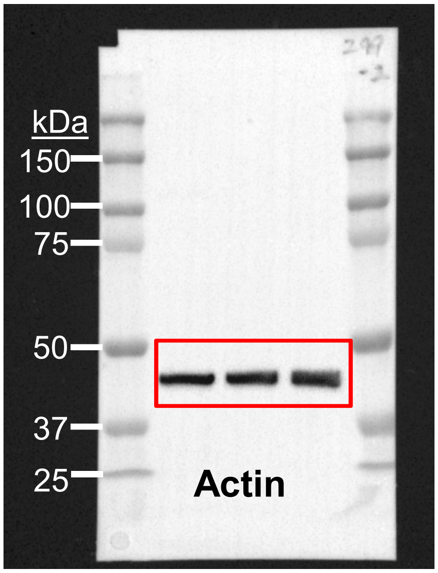

Supplementary Figure S8 (related to Figure 3G LNCaP)
Whole Western blots are shown. Intensity of each band is normalized to that of actin and ratios are indicated on the figure. Red rectangles indicate the proteins of interest.

Supplementary Figure S9
(related to Figure 3G VCaP)

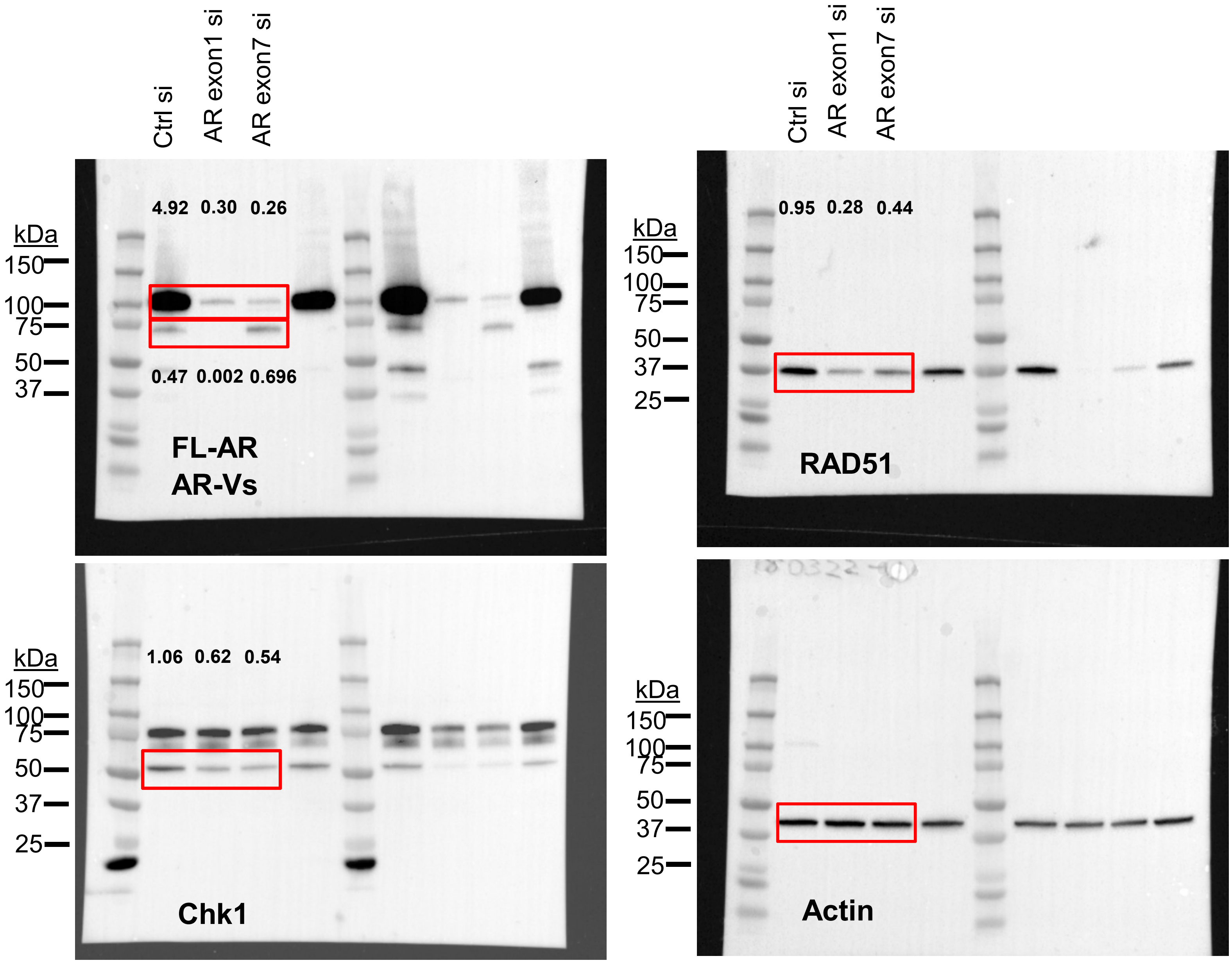

Supplementary Figure S9 (related to Figure 3G VCaP)
Whole Western blots are shown. Intensity of each band is normalized to that of actin and ratios are indicated on the figure. Red rectangles indicate the proteins of interest.

Supplementary Figure S10
(related to Figure 6A)

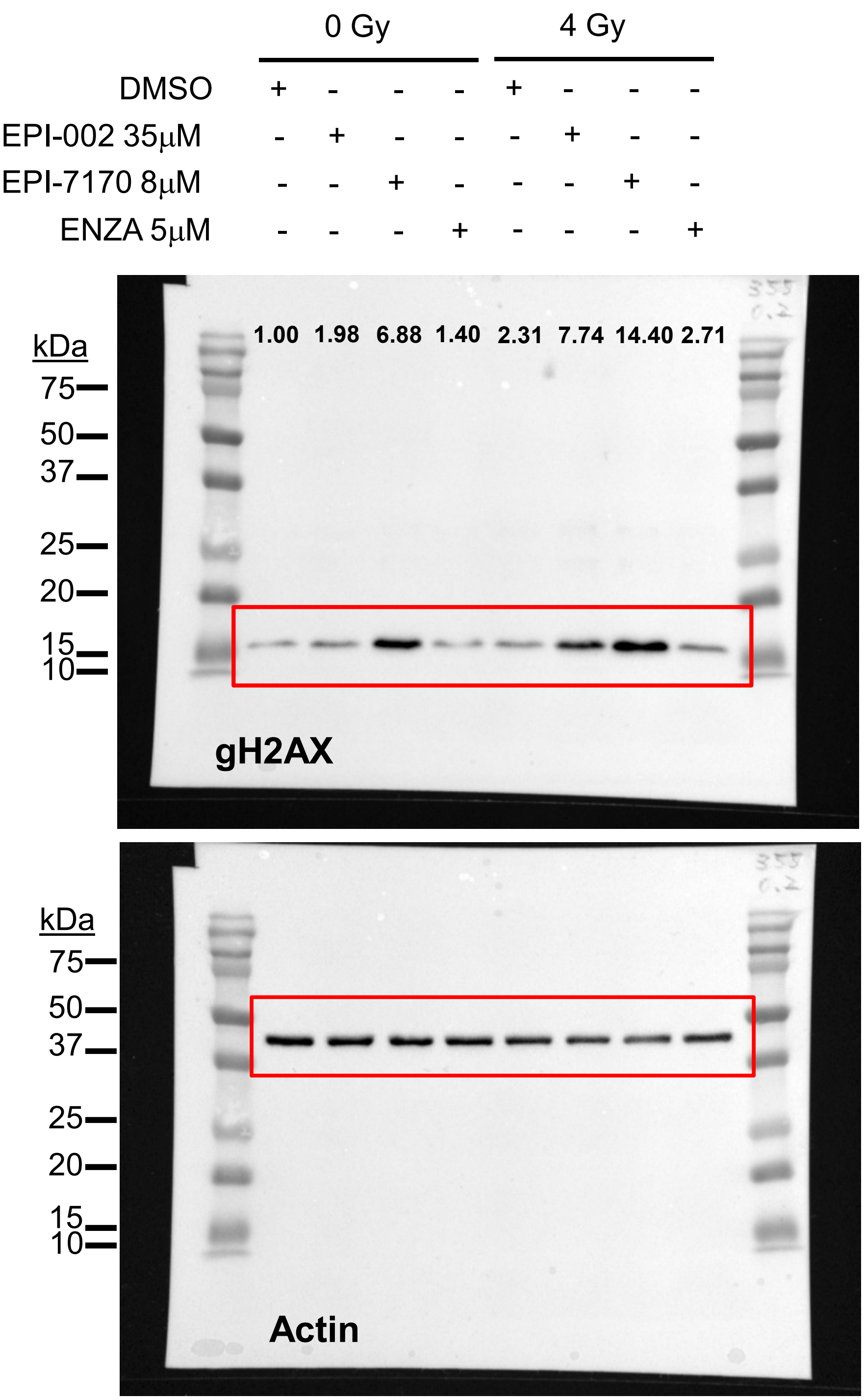

Supplementary Figure S10 (related to Figure 6A)
Whole Western blots are shown. Intensity of each band is normalized to that of actin and basal level of non-treated cells (DMSO control). Normalized ratios are indicated on the figure. Red rectangles indicate the proteins of interest.
